# Brain-derived neurotrophic factor but not beta-secretase 1, vascular endothelial growth factor, glial fibrillary acidic protein and interleukin-1β correlate with cognitive impairment in adult persons with epilepsy: a cross-sectional single-center study from India

**DOI:** 10.3389/fneur.2025.1540915

**Published:** 2025-04-11

**Authors:** Kamini Bhavsar, Manjari Tripathi, Jyotirmoy Banerjee, Arpna Srivastava, Nidhi, Shivam Pandey, Divya Vohora

**Affiliations:** ^1^Department of Pharmacology, School of Pharmaceutical Education and Research (SPER), Jamia Hamdard, New Delhi, India; ^2^Department of Neurology, All India Institute of Medical Science (AIIMS), New Delhi, India; ^3^Department of Biophysics, All India Institute of Medical Science (AIIMS), New Delhi, India; ^4^School of Chemical and Life Sciences (SCLS), Jamia Hamdard, New Delhi, India; ^5^Department of Biostatistics, All India Institute of Medical Science (AIIMS), New Delhi, India

**Keywords:** Montreal Cognitive Assessment (MoCA), brain-derived neurotrophic factor, beta secretase-1, vascular endothelial growth factor, glial fibrillary acidic protein, interleukin-1β

## Abstract

**Objectives:**

This study aims to evaluate cognitive impairment utilizing the Montreal Cognitive Assessment (MoCA) scale, while also exploring the correlation between cognitive impairment and various serum biomarkers, including Brain-derived neurotrophic factor (BDNF), Beta Secretase-1 (BACE1), Vascular Endothelial Growth Factors (VEGF), Glial fibrillary acidic protein (GFAP), and Interleukin-1 (IL-1β) in adults living with epilepsy.

**Methods:**

In this study, 74 participants aged between 18 and 50 years, who were visiting neurology outpatient consultations, were included. The cognitive assessment was executed using the MoCA test. Serum levels of BDNF, BACE1, VEGF, GFAP, and IL-1β were evaluated through ELISA in patients with and without cognitive impairments. To determine the association between MoCA scores and the biomarkers, both Spearman and Pearson correlation analyses, as well as linear regression, were conducted.

**Results:**

Among the 74 PWE, 61 exhibited cognitive impairment as determined by the MoCA assessment. Noteworthy alterations were detected across various MoCA subscales, encompassing visuospatial and executive functions, attention, language, abstraction, and delayed recall, with statistical significance established (*p* < 0.05). Furthermore, it was revealed that those in the cognitively impaired group presented with reduced serum BDNF levels (*p* < 0.05). It is important to highlight that no substantial differences were identified in the serum concentrations of BACE-1, VEGF, GFAP, and IL-1β. A moderate and statistically significant correlation was established between BDNF and the Total MoCA score (*p* < 0.05), in addition to BDNF’s relationship with Visuospatial & Executive function (*p* < 0.05). In the context of regression analysis, BDNF demonstrated a significant association to the Total MoCA score (*p* < 0.05), a connection that persisted as significant even when adjusted for confounding factors.

**Conclusion:**

We conclude that adult PWE in India demonstrate a significant cognitive impairment. Further, our findings indicate that BDNF may serve as a potential biomarker for evaluating cognitive impairment in adult PWE. Further longitudinal, prospective and multi-center studies are required to confirm the same.

## 1 Introduction

Persons with epilepsy (PWE) often experience cognitive impairment that manifests in various cognitive domains, such as attention, memory, executive functions, naming, visuospatial capabilities, and psychomotor speed (1). The International League Against Epilepsy (ILAE) reports that nearly half of those newly diagnosed with epilepsy, encompassing both children and adults, face cognitive or behavioral difficulties during evaluations. Considering this, the neuropsychological task force of ILAE has recommended that all newly diagnosed individuals undergo regular screening for such cognitive and behavioral concerns (2). Cognitive dysfunctions prevalent among those with epilepsy are deeply rooted in the condition’s underlying etiology, shaped by an array of factors including genetics, type of epilepsy, site and side of brain lesion, age at onset, and duration of epilepsy (3, 4) and the dynamic variables that include uncontrolled seizures, seizure frequency and severity, ictal as well as interictal transient focal epileptic discharges, adverse effects from antiseizure medications, and psychosocial variables (5, 6).

Additionally, the administration of antiseizure medications (ASMs) has been linked to cognitive decline (7, 8). While the older first-generation ASMs like phenytoin, carbamazepine, valproate, phenobarbital, and benzodiazepines are well-known to impair cognitive functions, among the second-generation ASMs, felbamate, gabapentin, topiramate, tiagabine, vigabatrin, zonisamide, pregabalin, rufinamide, brivaracetam, perampanel are implicated in cognitive impairment (9). Further, reports indicate that levetiracetam, lacosamide, oxcarbazepine, and lamotrigine can improve cognitive functions (10).

There are no established biomarkers for diagnosing cognitive impairment in PWE though studies have looked at the association of certain potential markers for cognitive impairment associated with epilepsy.

Brain-derived neurotrophic factor (BDNF) is classified within the neurotrophic family and is essential for several critical processes, including neuronal survival, synaptic plasticity, cell morphology determination, and the formation of memory (11). A decrease in serum BDNF levels has been linked to cognitive impairments across a range of disorders including Alzheimer’s disease (12) and MCI (13, 14). To the best of our knowledge, there are only two studies - one from Taiwan and another one from Indonesia that have investigated the link between BDNF and cognitive deficits in individuals with epilepsy. The findings from Taiwan indicated that lower serum BDNF levels were significantly associated with reduced scores in verbal memory and design fluency in temporal lobe epilepsy patients (12) while the Indonesian study showed a positive correlation between BDNF levels and cognitive functions in epilepsy patients treated with valproate and phenytoin (15).

Beta secretase 1, also known as beta-site amyloid precursor protein (APP) cleaving enzyme (BACE1), is an essential enzyme that facilitates the generation of amyloid-beta. Studies have shown that the activity of BACE1 is significantly elevated in the brain tissue (16, 17) and cerebrospinal fluid (CSF) (13) of patients suffering from mild cognitive impairment and sporadic Alzheimer’s disease. Furthermore, elevated serum levels of BACE-1 have been associated with cognitive decline in Alzheimer’s disease (14). Despite emerging evidence linking Alzheimer’s disease with epilepsy (18, 19), there is a notable absence of studies examining the modulation of BACE-1 in individuals with epilepsy. A preclinical study demonstrated that BACE1-null mice exhibit spontaneous seizures and increased seizure susceptibility (20, 21). There is also evidence of an association between BACE1 gene polymorphisms and focal seizures, particularly in males, indicating a potential genetic risk factor for epilepsy (22).

Vascular endothelial growth factor (VEGF) is a key angiogenic factor that primarily regulates the proliferation of endothelial cells. It plays a significant role in neuronal survival, neuroprotection, regeneration, growth, differentiation, and axonal outgrowth (23). Elevated levels of VEGF have been linked to a slower rate of cognitive decline in older individuals (24). Preclinical research has demonstrated that enhancing VEGF/VEGFR signaling can aid cognitive recovery post-epileptic seizures by fostering neurogenesis in the dentate gyrus (25, 26). Other preclinical evidence too suggests a role of VEGF in seizure-associated cognitive functions. For instance, VEGF positively contributed to the initial stages of neurogenesis and alleviated cognitive deficits following seizures in animals (26).

The glial fibrillary acidic protein (GFAP) is a cytoskeletal protein specific to astrocytes, functioning as an indicator of abnormal astrocytic activation and proliferation resulting from neuronal injury, a process referred to as astrogliosis. Under normal physiological conditions, astrocytes are crucial for memory consolidation; but when their regulation is impaired, they secrete GFAP, which has been linked to detrimental memory outcomes in animal studies. Moreover, GFAP levels in the blood have been found to have an inverse relationship with cognitive function (27). According to a recent meta-analysis, individuals diagnosed with Alzheimer’s disease exhibit significantly higher levels of GFAP in their cerebrospinal fluid when compared to cognitively unimpaired individuals (28). Despite being associated with memory decline, its role in cognitive impairment related to epilepsy has yet to be explored. It has been reported that elevated levels of GFAP, a marker of astrocytic activation, are often observed in patients with epilepsy and is associated with neuroinflammation, which can possibly impair cognitive functions (29). Further, GFAP expression is also elevated in cases of drug-resistant epilepsy linked to focal cortical dysplasia, highlighting its involvement in neurodegenerative processes (30). However, the lack of a significant correlation between GFAP levels and seizure characteristics suggests that its role may be more aligned with general neuroinflammatory responses rather than specific seizure types (31).

IL-1β is a key inflammatory mediator that contributes to the pathogenesis of epilepsy, particularly in drug-resistant cases. Studies have shown that the expression of IL-1β is increased in epilepsy, which correlates with a rise in both the frequency and intensity of seizures (32–34). Further, epilepsy associated neuroinflammation is characterized by elevated levels of IL-1β, and the same been implicated in both seizure activity and cognitive decline (35, 36).

This research focuses on evaluating cognitive impairment in individuals with epilepsy (PWE) using the Montreal Cognitive Assessment (MoCA) scale. Our preference for MoCA over the mini-mental state examination (MMSE) stems from its enhanced sensitivity and reliability, as well as its ability to detect cognitive diversity in neurological disorders (37, 38). Further, we investigated the relationship between cognitive impairment and the possible roles of serum BDNF, BACE1, VEGF, and GFAP as potential biomarkers for cognitive impairment in people with epilepsy. While these biomarkers have been previously evaluated for cognitive impairment, their specific associations with cognitive impairment in PWE remained unexamined.

## 2 Methodology

### 2.1 Study design, setting, and subjects

This observational, cross-sectional study was carried out at the Epilepsy Outpatient Department (OPD) of All India Institute of Medical Sciences (AIIMS) in New Delhi from November 2022 to February 2023. A random sampling method was used for the recruitment of patients. We successfully recruited 74 participants, consisting of 42 males and 32 females, The criteria mandated that participants be aged 18 to 50 years, diagnosed with epilepsy per the International League Against Epilepsy guidelines, have experienced at least one seizure within 12 months, and possess at least 5 years of education and understanding of the study consent form or if not, then a caregiver who could provide the information required for psychometric assessments. The study excluded individuals with a history of severe neurological conditions aside from epilepsy, as well as those with uncontrolled and untreated diabetes or thyroid disorders. Patients with a history of drug or alcohol abuse, those experiencing clinically unstable or life-threatening medical issues, or who had undergone surgery in the past year were not included. Those with a medication history involving psychoactive or central nervous system depressants, as well as patients exhibiting abnormal renal or liver function, were also omitted from the study.

### 2.2 Study variables

Cognitive impairment served as the dependent variable in this analysis, evaluated using the MoCA, which was treated as a dichotomous variable (Yes/No). The independent variables encompassed socio-demographic characteristics such as age, gender, marital status, and occupation, along with clinical factors including a history of head trauma, family history of seizures, previous use of alcohol and narcotics, and a history of severe mental illness and seizure-related factors were considered, including the type of seizure or epilepsy, frequency of seizures, age at which seizures began, duration of the seizure disorder, and both past and current anti-seizure medications.

### 2.3 Assessment of cognitive functions through MoCA

To evaluate cognitive functions, all patients completed the MoCA questionnaire (version 7.1; Supplementary Figure S3) after securing the required copyright permissions and completing the relevant training certification.^1^ The cognitive functions assessed included Visuospatial & Executive Naming, Attention, Language, Abstraction, Delayed Recall, and Orientation. PWE were categorized into cognitively impaired and cognitively unimpaired groups according to their MoCA scores. Among those identified as cognitively impaired, patients were further classified into mild (MoCA score 18–25), moderate (MoCA score 11–17), and severe (MoCA score less than 10) categories based on the severity of their condition.

The following procedure was followed:

1. Alternating Trail Making: Participants were directed to “Connect a line from a number to a letter in sequential order.” Each correctly executed pattern earned one point: 1-A, 2-B, 3-C, 4-D, 5-E.
2. Visuo-constructional Skills (Cube): Participants were instructed to accurately reproduce the drawing of the cube in the area designated below. One point was given for the successful completion of a drawing.
3. Visuo-constructional Skills (Clock): Participants were instructed by the investigator: “To create a clock, ensure all numbers are included, and set the time to 10 min past 11.” Scoring was based on three specific criteria: (1) The clock should have a circular shape, (2) All numbers must be displayed in the correct order and located in the approximate quadrants of the clock face, with the possibility of placing them outside the circular shape, and (3) The hour hand must be shorter than the minute hand, properly indicating the specified time.
4. Naming: While pointing to each illustration, participants were instructed to provide the name of the animal shown. They earned one point for each animal named correctly: (1) lion, (2) rhinoceros or rhino, and (3) camel.
5. Memory: The investigator recited a series of five words, each at a rate of one per second while guiding participants to commit them to memory for immediate and later recall. After completing the list, participants were prompted to recall as many words as possible. Upon finishing the second trial, participants were made aware that they would be required to remember the words. There were no points assigned for the trials.
6. Attention.

Forward Digit Span: The participants received the following directive: “I will announce a series of numbers, and once I have finished, please repeat them back to me exactly as I stated them.” The five-number sequence was to be read at a pace of one digit per second.

Backward Digit Span: Participants were instructed as follows: “I will now state several numbers, and after I finish, you must repeat them to me in reverse order.” The three-number sequence was delivered at a speed of one digit per second, with participants earning one point for each sequence they accurately repeated.

Vigilance: I reviewed the sequence of letters at a pace of one per second, following the instruction: “I will read a series of letters. Please tap your hand once each time I mention the letter A. Do not tap your hand for any other letters.” A score of one point is awarded for making zero to one mistake.

Serial 7 s: The participants received directions to start with 100 and repeatedly subtract seven from their current total until they were told to cease. This task is evaluated on a scale of 3 points: 0 points for failing to make any correct subtractions, 1 point for achieving one correct subtraction, 2 points for two to three correct subtractions, and 3 points for successfully performing four or five correct subtractions.

1. Sentence repetition: Following the investigator’s reading participants were directed to accurately repeat the phrase “I only know that John is the one to help today” following the sentence “The cat always hid under the couch when dogs were in the room.” They were awarded one point for each accurate repetition.
2. Verbal fluency: The participants were tasked with listing as many words as they could that begin with the letter ‘F’, with the stipulation that proper nouns and numbers were not permitted. They earned one point for successfully naming 11 or more words within a 60-s period.
3. Abstraction: The participants were instructed to identify the commonalities between each set of words. Initially, they were asked to discuss the similarities between a train and a bicycle, followed by an explanation of how a ruler and a watch are similar. Acceptable responses included: Trains and bicycles serve as modes of transportation, facilitating travel and trips. Rulers and watches function as measuring tools. Each correctly paired response earned one point.
4. Delayed recall: Participants were requested to enumerate as many words as they could recall from the previously read list. Each word remembered independently, without any hints, earned them 1 point (see Footnote 1).

### 2.4 Blood sampling and biochemical analysis through ELISA

The time elapsed between sample collection and processing was immediate. Peripheral blood samples of 10 mL were collected via venipuncture into Vacutainer® tubes that included Clot Activator. Following a 30-min incubation period at room temperature, the samples were centrifuged at 4 degrees Celsius at a speed of 1,500 rpm (RCF 290). The serum was subsequently separated into single-use aliquots and stored at −80 degrees Celsius. The analysis of serum concentrations for BDNF, BACE-1, VEGF, GFAP, and IL-1β was conducted in duplicates utilizing Krishgene BioSystems Enzyme-linked immunosorbent assay (ELISA) kits, adhering to the procedures specified in the standardized manual. The photometric evaluation of the resulting color product was conducted using a Multiscan Go microplate reader within 1 h at a wavelength of 450 nm. The concentrations and standard curves for the biomarkers were determined through the AAT Bioquest software, employing the Four Parameter Logistic (4PL) curve calculator provided by the company.

### 2.5 Sample size determination

To determine the sample size, the single population proportion formula was employed. The prevalence (p) of 72% (0.72), sourced from research by Samarasekera SR et al. (65), was considered, along with a confidence level of 1.96 (95%) and a precision or absolute error of 10%. Consequently, the calculated sample size amounted to 74.

### 2.6 Statistical analysis

The statistical analyses were conducted utilizing STATA14.0 software. Categorical socio-demographic and clinical data were expressed in terms of frequency (%). Group comparisons for categorical variables were carried out using Fisher’s exact test. The MoCA scores, along with their domain and biomarker variables, are reported as Mean ± SD or Median (min, max). To determine the differences between the two groups with normally distributed data, we utilized the independent two-sample t-test. For data that did not meet the normality assumption, the Wilcoxon Rank Sum test (Mann Whitney U) was conducted. we evaluated the correlation between biomarkers and the MoCA, as well as its domains, by employing the Spearman test for non-normally distributed variables and Pearson’s test for those that were normally distributed. A linear regression analysis was conducted to explore the relationship between biomarkers and Total MoCA. Additionally, multiple linear regression analysis was performed to assess the independent association of each identified confounding factor with BDNF levels. A *p*-value of less than 0.05 was regarded as statistically significant. The diagnostic strength and the optimal cut-off value were evaluated through the area under the ROC (AUROC) curve and sensitivity analysis, respectively.

### 2.7 Standard protocol approvals, registrations, and patients informed consent

The clinical study received approval from the Institutional Ethics Committee of the All India Institute of Medical Sciences (AIIMS) under approval number 757/07, dated October 2022, and from the Jamia Hamdard Institutional Ethics Committee. The research was conducted in compliance with the Declaration of Helsinki and the ICMR Ethical Guidelines for Biomedical Research Involving Human Participants, 2017. All subjects were adequately informed about the study and provided their informed consent, both orally and in writing, in either English or Hindi, based on their preference.

## 3 Results

### 3.1 Socio-demographic and clinical characteristics

Table 1 presents a comparison of socio-demographic and clinical parameters between the cognitively impaired and cognitively unimpaired groups among the study participants. A greater proportion of participants were early adults (ages 18–30), with males constituting 51.35% of the sample, outnumbering female. The predominant education level among participants was college or higher. The unemployment rate was significant at 27.03, and 54.05% of the participants were unmarried. Notable statistical significance was found in the areas of education (*p* = 0.049) and gender (*p* = 0.0005) between the cognitively impaired and unimpaired groups.

**Table 1:** Demographic and clinical characteristics of adult persons with epilepsy assessed through MoCA (*n* = 74).

Variables	N (%)	Cognitively impaired (*n* = 61) N (%)	Cognitively unimpaired (*n* = 13) N (%)	*p*-value
Age (18–50 years)				
18–30	68 (68.92)	42 (68.85)	9 (69.23)	0.444
31–45	21 (28.38)	18 (29.51)	3 (23.08)	
>45	2 (2.70)	1 (1.64)	1 (7.69)	
Gender				
Male	38 (51.35)	36 (59.02)	2 (15.38)	**0.0005**
Female	36 (48.65)	25 (40.98)	11 (84.62)	
Educational level				
Primary school	9 (12.16)	9 (14.75)	0	**0.049**
Junior Secondary	16 (21.62)	15 (24.59)	1 (7.69)	
Higher Secondary	13 (17.57)	12 (19.67)	1 (7.69)	
College level or above	36 (48.65)	25 (40.98)	11 (84.62)	
Occupation				
Student	17 (22.97)	11 (18.03)	6 (46.15)	0.446
Government	1 (1.35)	1 (1.64)	0	
Private	11 (14.86)	10 (16.39)	1 (7.69)	
Self-employment	11 (14.84)	9 (14.75)	2 (15.38)	
Homemaker	14 (18.92)	12 (19.67)	2 (15.38)	
Unemployment	20 (27.03)	18 (29.51)	2 (15.38)	
Marital status				
Married	34 (45.95)	30 (49.18)	4 (30.77)	0.359
Unmarried	40 (54.05)	31 (50.82)	9 (69.23)	
Duration of illness (years)				
0–10	30 (40.54)	23 (37.70)	7 (53.85)	0.438
11–20	35 (47.30)	31 (50.82)	4 (30.77)	
21–30	9 (12.16)	7 (11.48)	2 (15.38)	
Type of Seizures/				
Epilepsy	38 (51.35)	30 (49.18)	8 (61.54)	1.000
Focal Generalized	23 (31.08)	19 (31.14)	4 (30.77)	
Focal + Generalized	10 (13.51)	8 (13.11)	2 (15.38)	
Unknown	3 (4)	3 (4.9)	0	
Seizure frequency				
Days	6 (8.11)	5 (8.20)	1 (7.69)	0.565
Monthly	48 (64.86)	41 (67.21)	7 (53.85)	
Yearly	20 (27.03)	15 (24.59)	5 (38.46)	
Therapy				
Polytherapy	61 (82.43)	10 (16.39)	1 (7.69)	0.676
Monotherapy	11 (14.84)	51 (83.61)	12 (92.31)	
Number of seizures				
1–10	72 (97.30)	59 (96.72)	13 (100)	1.000
11–20	0	0	0	
21–30	1 (1.35)	1 (1.64)	0	
>30	1 (1.35)	1 (1.64)	0	
Age of onset (years)				
1–10	18 (24.32)	16 (26.23)	2 (24.32)	0.699
11–20	36 (48.65)	28 (45.90)	36 (48.65)	
21–30	15 (20.27)	12 (19.67)	15 (20.27)	
> 30	5 (6.76)	5 (8.20)	5 (6.76)	
Duration of seizures				
1–59 s	24 (32.43)	18 (29.51)	6 (46.15)	0.514
1–15 min	44 (59.46)	38 (62.30)	6 (46.15)	
>15 min	6 (8.11)	5 (8.20)	1 (7.69)	

Values are presented as N (%), *N*, Frequency, the mean differences between the groups were analyzed using Fisher’s exact test. *p*-value < 0.05 is considered statistically significant and is depicted in bold.

Majority of participants experienced epilepsy for a duration ranging from 11 to 20 years, accounting for 47.30%. Approximately one-third of the patients (64.86%) reported a high frequency of seizures each month, while 27.03% of the individuals were either seizure-free or had no more than one seizure annually. A large proportion of patients were on a polytherapy (39) and only 12 on monotherapy. The most commonly prescribed ASMs on polytherapy were clobazam and levetiracetam followed by valproic acid, lacosamide and carbamazepine. Other drugs in polytherapy were oxcarbazepine, phenytoin, brivaracetam, topiramate, perampanel, clonazepam. The least prescribed drugs in polytherapy were zonisamide and phenobarbitone (Supplementary Tables S1, S2). Majority of patients had focal seizure (51.35%) followed by generalized seizure (31.08%), combined focal and generalized (13.51%) and unknown category of seizure (4%).

### 3.2 Cognitive assessment using MoCA

Table 2A, the findings from two-sample t-tests and the Wilcoxon rank sum (Mann–Whitney U) test for MoCA and its domains are illustrated. A *p*-value below 0.05 was regarded as statistically significant. Depending on how the data variables are distributed, the results for visuospatial and executive functions, language, abstraction, and delayed recall are reported as median (min, max). Whereas, naming, attention, orientation, and the overall MoCA score are expressed as mean ± standard deviation. Among the 74 patients with epilepsy (PWE), 61 individuals (82.43%) exhibited cognitive impairment, while 13 patients (17.56%) were classified as cognitively unimpaired according to the MoCA assessment. The subscales of MoCA, which include visuospatial and executive functions, attention, language, abstraction, delayed recall, and the overall MoCA score, demonstrated a statistically significant decline in the cognitively impaired group when compared to the cognitively unimpaired group (*p* = 0.0000, 0.0004, 0.0004, 0.0061, 0.0000, respectively).

**Table 2A:** Comparison of MoCA scores in cognitively impaired and unimpaired PWE (*n* = 74).

Mo MoCA	Cognitively impaired (*n* = 61) Mean ± SD Or Median (min, max)	Cognitively unimpaired (*n* = 13) Mean ± SD Or Median (min, max)	*p*-value
Visuospatial & executive	1 (0, 5)	4 (3, 5)	**0.0000** ^§^
Naming	2.43 ± 0.62	2.77 ± 0.44	0.0618^¶^
Attention	3.47 ± 1.95	5.53 ± 0.78	**0.0004** ^¶^
Language	1 (0,2)	2 (0,3)	**0.0004** ^§^
Abstraction	1 (0,2)	2 (1,2)	**0.0061** ^§^
Delayed recall	1 (0,6)	4 (4,5)	**0.0000** ^§^
Orientation	5.20 ± 1.67	6 ± 0	0.0891^¶^
MoCA total	16.30 ± 5.97	26.77 ± 1.16	**<0.001** ^¶^

Two sample t-tests (¶) and Wilcoxon rank sum (Mann–Whitney U) (§) tests were used, as appropriate. *p*-value < 0.05 is considered statistically significant and is depicted in bold. MoCA-Montreal cognitive assessment, SD, Standard deviation.

Table 2B indicates that the MoCA scale possesses strong internal consistency and reliability for assessing cognitive impairment in adult PWE, reflected by a Cronbach’s alpha of 0.8. The area under the ROC curve (Supplementary Figure S1) shows that the MoCA is an outstanding tool for assessing cognitive impairment in this population, with an AUROC value of 1.

**Table 2B:** Internal consistency assessment of MoCA and its subdomains (*n* = 74).

MoCA	Cronbach’s alpha
Visuospatial & executive	0.73
Naming	0.79
Attention	0.73
Language	0.8
Abstraction	0.78
Delayed recall	0.78
Orientation	0.76

### 3.3 Assessment of serum biomarkers

In Table 3, the analysis compares the levels of BDNF (pg/ml), BACE1 (ng/ml), VEGF (pg/ml), GFAP (ng/ml), and IL-1β (pg/ml) between groups with cognitive impairment and cognitively unimpaired, utilizing two-sample t-tests and the Wilcoxon rank sum (Mann–Whitney U) test as appropriate. In the group of cognitively impaired PWE, BDNF (pg/ml) levels were significantly reduced (*p* = 0.0000). However, serum levels of BACE1 (ng/ml), VEGF (pg/ml), GFAP (ng/ml), and IL-1β (pg/ml) did not show any significant differences. According to the AUROC analysis (Supplementary Figure S2), BDNF is recognized as a reliable biomarker for the assessment of cognitive impairment in this group, with an AUROC value of 0.8550. The sensitivity/specificity analysis revealed that the optimal cutoff point is 132.07 pg./mL.

**Table 3:** Comparison of biomarkers in cognitively impaired and unimpaired groups (*n* = 74).

Biomarkers	Cognitively impaired (*n* = 61) Mean ± SD Or Median (min, max)	Cognitively unimpaired (*n* = 13) Mean ± SD Or Median (min, max)	*p*-value
BDNF pg./ml	129.01 ± 9.35	158.17 ± 26.78	**0.0000** ^¶^
BACE-1 ng/mL	0.48 ± 0.19	0.44 ± 0.13	0.4760^¶^
VEGF pg./ml	119.93 ± 26.58	118.55 ± 12.83	0.8564^¶^
IL-1β pg./ml	11.91 (1.64, 48.18)	8.16 (2.86, 55.21)	0.7172^§^
GFAP ng/ml	5.96 (3.24, 52.37)	5 (2.71, 9.01)	0.0896^§^

Two sample t-tests (¶) and the Wilcoxon rank sum (Mann–Whitney U) (§) test were performed, as appropriate. *p* value < 0.05 is considered statistically significant and is depicted in bold. SD, Standard Deviation; BDNF, Brain-derived neurotrophic factor; BACE-1, Beta Secretase-1; VEGF, Vascular Endothelial Growth Factors; GFAP, Glial fibrillary acidic protein; IL-1β, Interleukin-1β. pg, picogram, ng, nanogram.

### 3.4 Correlation analysis between biomarkers and cognitive performance

The Spearman analysis revealed a moderate and significant correlation (*p* = 0.0028) between BDNF and Total MoCA, as presented in Table 4A. Furthermore, Table 4B highlights the correlations between the MoCA subdomains and various biomarkers, using both Spearman and Pearson’s tests. Findings indicated a moderate and significant correlation (*p* = 0.0005) between BDNF and visuospatial and executive functions. Further, a mild but significant connection was noted between BDNF and the areas of naming, attention, and abstraction (*p* = 0.039, 0.04, 0.03, respectively).

**Table 4A:** Association between MOCA Total and biomarkers (*n* = 74).

		MOCA Total	BDNF	IL-1β	GFAP	BACE-1	VEGF
MOCA Total	Correlation	1	0.34	−0.03	−0.13	−0.1	−0.07
	P		**0.0028** ^¥^	0.784^€^	0.261^€^	0.38^€^	0.543^€^
BDNF	Correlation	0.34	1	0.02	0.14	0.11	0.06
	P	**0.0028** ^¥^		0.863	0.221	0.348	0.63
IL-1β	Correlation	−0.03	0.02	1	−0.08	0.11	0.11
	P	0.784^€^	0.863		0.509	0.349	0.34
GFAP	Correlation	−0.13	0.14	−0.08	1	0	−0.13
	P	0.261^€^	0.221	0.509		0.978	0.266
BACE-1	Correlation	−0.1	0.11	0.11	0	1	0.35
	P	0.38^€^	0.348	0.349	0.978		0.002
VEGF	Correlation	−0.07	0.06	0.11	−0.13	0.35	1
	P	0.543^€^	0.63	0.34	0.266	0.002	

Spearman (€) and Pearson’s (¥) correlation was performed. *p*-value < 0.05 is considered statistically significant and is depicted in bold. MoCA, Montreal cognitive assessment; BDNF, Brain-derived neurotrophic factor; BACE-1, Beta Secretase-1; VEGF, Vascular Endothelial Growth Factors; GFAP, Glial fibrillary acidic protein; IL-1β, Interleukin-1β.

**Table 4B:** Association between MOCA sub-domains and biomarkers (*n* = 74).

MoCA sub-domains		BDNF	BACE-1	VEGF	IL-1β	GFAP
Visuospatial & Executive	Correlation	0.39	−0.15	−0.1	0.04	−0.0983
	P	**0.0005** ^¥^	0.189^€^	0.408^€^	0.755^€^	0.404^€^
Naming	Correlation	0.24	0.09	0.09	0.14	−0.02
	P	**0.039** ^¥^	0.446^€^	0.43^€^	0.24^€^	0.864^€^
Attention	Correlation	0.23	−0.06	0.09	−0.14	−0.08
	P	**0.04** ^¥^	0.623^€^	0.449^€^	0.222^€^	0.506^€^
Language	Correlation	0.21	0.27	0.14	0.04	−018
	P	0.072^€^	0.021^€^	0.251^€^	0.757^€^	0.115^€^
Abstraction	Correlation	0.24	−0.03	−0.07	0.16	0
	P	**0.03** ^¥^	0.777^€^	0.555^€^	0.183^€^	0.975^€^
Delayed recall	Correlation	0.24	−0.22	−0.19	−0.09	−0.16
	P	0.06^¥^	0.062^€^	0.114^€^	0.423^€^	0.174^€^
Orientation	Correlation	0.06	0.02	−0.17	−0.01	−0.03
	P	0.60^¥^	0.863^€^	0.137^€^	0.907^€^	0.78^€^

Spearman’s (€) and Pearson’s (¥) correlation was performed. *p*-value < 0.05 is considered statistically significant and is depicted in bold. BDNF, Brain-derived neurotrophic factor; BACE-1, Beta Secretase-1; VEGF, Vascular Endothelial Growth Factors; GFAP, Glial fibrillary acidic protein; IL-1β, Interleukin-1β.

### 3.5 Regression analysis of biomarkers and total MoCA

Table 5 illustrates the significant association between BDNF levels and Total MoCA, indicated by linear regression analysis (*p* = 0.003), confidence interval (0.228, 0.516). To assess the association while controlling for confounding factors, a multiple linear regression analysis was carried out, considering variables such as age, gender, marital status, education level, occupation, seizure frequency, days with seizures, age of onset, duration of the illness, seizure duration, family history, and therapeutic interventions. A one-unit change in the MOCA total variable results in a 0.98 unit change in the BDNF variable, with a *p*-value of 0.032, indicating a significant relationship between MOCA total and BDNF levels (pg/ml; Table 6). In contrast, BACE-1, VEGF, GFAP, and IL-1β did not exhibit significant correlations with the total MoCA score (Table 5).

**Table 5:** Linear regression analysis of MOCA total and biomarkers (*n* = 74).

MOCA total biomarkers	Unadjusted coefficients	Adjusted coefficients				95% confidence interval for B	
	B	Beta	Standard error	T	*p*	lower bound	upper bound
(Constant)	117.83		5.62	20.96	<0.001	106.62	129.04
BDNF	0.1303	0.1303688	0.0421	3.09	**0.003**	0.228	0.516
BACE-1	−1.2944	−0.124	4.307	−1.102	0.274	−13.772	3.465
VEGF	−0.0326	0.82	0.075	0.697	0.488	−0.103	0.022
IL-1β	0.0521	0.036	0.072	0.319	0.751	−0.104	0.188
GFAP	−0.0439	−0.064	0.498	−0.570	0.570	−7.231	20.761

*p-value < 0.05 is considered statistically significant and is depicted in bold. MoCA, Montreal cognitive assessment; BDNF, Brain-derived neurotrophic factor; BACE1, Beta Secretase-1; VEGF, Vascular Endothelial Growth Factors; GFAP, Glial fibrillary acidic protein; IL-1β, Interleukin-1β. BDNF, BACE-1, VEGF, IL1β and GFAP as independent variables and MoCA as dependent variables.*

**Table 6:** Clinical and sociodemographic correlates of BDNF (*n* = 74).

BDNF	Unstandardized coefficients	Standardized coefficients				95% confidence interval for B	
	B	Beta	Standard error	t	*p*	lower bound	upper bound
(Constant)	126.81		15.19	8.35	<0.001	96.43	157.18
Age	−0.131	−0.7408735	0.68	−1.08	0.283	−2.11	0.63
Gender	9.054	7.138283	4.59	1.56	0.125	−2.04	16.31
Marital status	3.838	3.31685	6.32	0.53	0.601	−9.32	15.95
Education	2.831	−1.486931	2.702	−0.55	0.584	−6.89	3.92
Occupation	−0.126	0.268668	1.302	0.21	0.837	−2.34	2.87
No. of seizures	0.0461	0.039435	0.472	0.08	0.934	−0.9	0.98
Seizure frequency	−0.002	−0.0031644	0.008	−0.42	0.679	−0.02	0.01
Age of onset	−0.1148	0.4487735	0.568	0.79	0.433	−0.69	1.59
Duration of illness	0.140	0.7331407	0.619	1.18	0.241	−0.5	1.97
Duration of seizure	−5.80e-06	0.0001958	0.001	0.14	0.892	0	0
Family history	6.231	6.2218	6.654	0.94	0.354	−7.09	19.53
Therapy	7.742	3.779575	6.4	0.59	0.557	−9.026	16.58
MOCA Total	0.90	0.97896	0.45	2.2	**0.032**	0.09	1.87

Multiple linear regression analysis was performed. Adjusted for confounding variables age, gender, marital status, education, occupation, number of seizures, seizure frequency, age of onset, duration of illness, duration of seizure, family history, and therapy. *p*-value < 0.05 is considered statistically significant and is depicted in bold.

### 3.6 Association between demographic variables and total MoCA

Table 7A depicts various demographic variables, such as education, gender, age, marital status, and occupation, which are analyzed through Pearson’s correlation analysis (Table 5). A significant positive correlation (*p* = 0.000) was found between education and total MoCA scores. Significant associations were also observed between gender, marital status, and total MoCA (*p* = 0.0217, 0.0224, 0.0052, respectively). The findings presented in Table 7B reveal a strong correlation between education and total MoCA scores, after adjusting for confounding variables through multiple linear regression analysis (*p* = 0.000). The findings indicate that education is significantly associated with cognitive impairment in PWE. A lower educational background may serve as a significant predictor of cognitive impairment in PWE.

**Table 7A:** Association between potential factors affecting cognitive impairment and total MoCA (*n* = 74).

		Total MoCA
Education	Correlation	0.6536
	P	**0.0000**
Gender	Correlation	0.2665
	P	**0.0217**
Therapy	Correlation	0.1273
	P	0.2797
Age	Correlation	0.0177
	P	0.8811
Marital status	Correlation	0.2651
	P	**0.0224**
Occupation	Correlation	−0.3216
	P	**0.0052**

Spearman’s correlation analysis was performed. *p*-value < 0.05 is considered statistically significant and is depicted in bold. MoCA, Montreal cognitive assessment.

**Table 7B:** Association between potential factors affecting cognitive impairment and Total MoCA (*n* = 74).

Total MoCA model	Unstandardized coefficients		Standardized coefficients	95.0% Confidence interval for B	
	B	*p*-value	Beta	Lower bound	Upper bound
Education	3.546177	**0.000**	4.022127	2.927887	5.116366
Gender	0.7568464	0.558	3.577485	0.5381501	6.616821
Therapy	0.9784683	0.577	−3.44066	−1.993317	6.795626
Age	−0.0989918	0.392	0.0167038	−0.2050346	0.2384421
Marital status	3.406775	0.050	3.569118	0.5196335	6.618602
Occupation	−0.229703	0.525	−1.149627	−1.944817	−0.3544362

Multiple linear regression analysis was performed. *p*-value < 0.05 is considered statistically significant and is depicted in bold.

## 4 Discussion

The central aim of our investigation was to assess cognitive impairment and to analyze the relationship between specific biomarkers - BDNF, BACE1, VEGF, GFAP and IL-1β and cognitive dysfunction in adults PWE. Our findings indicated a significant cognitive impairment in adult PWE and reduced serum BDNF in cognitively impaired persons. Further, we observed a positive association between BDNF level and Total MoCA which remained significant even after adjusting for possible confounders.

While the Mini-Mental State Examination (MMSE) is a common tool employed by physicians for general cognitive screening (40), our study opted for the Montreal Cognitive Assessment (MoCA) to evaluate cognitive impairment in persons with epilepsy (PWE). This choice is supported by numerous studies indicating that MoCA demonstrates greater sensitivity than the MMSE in detecting mild cognitive impairment (MCI) (41, 42). Research has shown that MoCA accurately captures the spectrum of MCI severity compared to MMSE in affected patients (43). Furthermore, MoCA has been recognized as a more comprehensive assessment tool, offering superior efficacy and specificity for evaluating cognitive functions in PWE relative to MMSE (44). In our investigation, out of 74 PWE, 61 individuals (82.4%) were identified as cognitively impaired according to MoCA.

Our findings indicate a significant reduction in total MoCA scores, with marked differences observed in cognitive functions such as visuospatial and executive abilities, attention, language, abstraction, delayed recall, and memory. Conversely, the parameters of naming and orientation did not show significant variation. This outcome is consistent with a multitude of studies that have reported decreased MoCA scores in PWE (41, 45, 46). Specifically, in individuals over 15 years old suffering from cryptogenic epilepsy, the MoCA subscales for visuospatial and executive functions, as well as naming, attention, language, abstraction, delayed recall, and orientation, were found to be significantly lower than those of cognitively healthy individuals (41). An additional study examining patients with generalized seizures revealed that these individuals had significantly lower scores on the MoCA, especially in the areas of executive functions and delayed recall, when compared to those suffering from psychogenic non-epileptic seizures (45). Moreover, research indicated that epilepsy patients presented with lower overall MoCA scores, as well as deficits in the subscales of language, naming, delayed recall, and attention, relative to healthy individuals (46). In the Indian demographic, evaluations of cognitive deficits among individuals with epilepsy utilizing the MoCA revealed 100% incidence of cognitive impairment (47). Furthermore, a notable MoCA score in epilepsy patients demonstrated a significant decline of 81.57% (48). Additionally, a study by Nathan R. in 2015 corroborated these findings, indicating prevalence of cognitive impairment in epilepsy patients (49).

With respect to the seizure types, TLE is strongly associated with memory deficits, particularly due to its impact on the hippocampus and related structures. This association is often aggravated by conditions such as hippocampal sclerosis, early onset, and recurrent seizures. TLE is associated with more pronounced cognitive impairments in memory and language functions and verbal abilities (18, 50). Those with generalized epilepsy are likely to experience less severe cognitive impairments than individuals with temporal lobe epilepsy (TLE), particularly in areas such as attention, executive functioning, and processing speed. In our study, we could not find any statistically significant differences in cognitive impairment with respect to the different seizure types (focal, generalized and temporal lobe epilepsy) which could be due to smaller sample size for each seizure type.

It has been previously established that education is a substantial risk factor for cognitive decline among individuals with epilepsy (1). Our study revealed that participants classified as cognitively impaired tended to have lower educational qualifications. Furthermore, multiple linear regression analysis identified a robust positive correlation (*r* = 0.6536) between education levels and total MoCA scores. These findings resonate with the research conducted by Ziari et al., which demonstrated that reduced educational level significantly predicted cognitive impairment via MoCA assessments in patients with generalized seizures (51). In a similar vein, another study noted that elevated education levels serve as a protective factor for cognitive health in individuals with seizures (1), while the lower educational level was significantly linked to cognitive impairment in epilepsy patients (52). However, the association might be confounded by other socioeconomic factors, such as income and access to healthcare which were not explored in the present work and is a limitation in this work.

BDNF is recognized as the predominant growth factor in the central nervous system, primarily associated with neuronal plasticity and the facilitation of memory performance (53). Our research indicates a significant decline in BDNF concentrations within the cognitively impaired group compared to those who are cognitively unimpaired among adult individuals with epilepsy (PWE). Furthermore, we discovered a moderate and statistically significant correlation between BDNF levels and the overall MoCA score. This correlation remained robust even after adjusting for potential confounding variables such as age, gender, marital status, educational background, occupation, seizure frequency, seizure days, age at onset, duration of the illness, seizure duration, family history, and treatment modalities. To the best of our understanding, our research represents the first report linking MoCA scores with BDNF levels in PWE. Our findings resonate with a Taiwanese study that identified a similar correlation using the MMSE scale (12).

Through our sub-scale analysis, we discerned a relationship between BDNF and various MoCA sub-scales, including visuospatial and executive function, naming, attention, and abstraction. BDNF is widely regarded as a significant biomarker in the realm of cognitive impairment, with numerous studies consistently demonstrating diminished BDNF levels in patients exhibiting cognitive deficits associated with mild cognitive impairment (MCI). A comprehensive cross-sectional observational study examining serum BDNF levels in elderly individuals with MCI revealed a significant association between reduced serum BDNF and reduced cognitive test performance (54). Similarly, another investigation highlighted a correlation between lowered serum BDNF levels and cognitive deficits in MCI patients (55). Furthermore, the severity of cognitive impairment in both Alzheimer’s Disease (AD) and MCI patients was found to be linked to lower serum BDNF levels (56). Notably, BDNF levels exhibited a positive correlation with the MMSE scores in MCI patients, establishing it as a promising biomarker for clinical diagnosis and therapeutic assessment in this population (57).

The effect of seizures and epilepsy on BDNF remain controversial. Sartoriu et al. suggested that BDNF reduction is associated with increased seizure frequency and severity (58) although some studies indicate that BDNF levels may rise after seizures (59) complicating the correlation. Further, ASMs may affect BDNF differently. In a preclinical study, it was reported that the administration of perampanel, lacosamide, and their combination in experimental model of temporal lobe epilepsy in adult Wistar rats significantly elevated the hippocampal levels of BDNF (60). On the contrary, another investigation in rats reported levetiracetam to abolish kindling-induced elevation of BDNF levels (39). In our study, as majority of the patients were on polytherapy, it is difficult to ascertain the effect of individual ASM on serum level of BDNF and other biomarkers. Hence, further research using drug monotherapy can confirm the exact relationship between individual ASM and BDNF.

We did not observe any significant difference in the levels of BACE-1, VEGF, GFAP, and IL-1β between cognitively impaired and unimpaired groups. Further, no association was observed between the MoCA score with these biomarkers investigated. Thus, though elevated BACE-1 levels are found to be increased in MCI patients (17, 61). We did not find any such alteration in cognitive impairment associated with epilepsy patients. Similarly, serum or plasma VEGF levels are reported to be significantly lowered in MCI patients (62, 63). For GFAP, blood levels were reported to inversely correlate with cognition (27). Higher plasma GFAP levels were associated with lower executive, visual memory, language, and visuospatial scores (64). In our findings, no significant differences were, however, observed in GFAP levels in cognitively impaired and unimpaired seizure patients. We also measured IL-1β levels as the same is associated with cognitive impairment in various diseases. However, we did not find a significant association between IL-1β and seizure-associated cognitive impairment. While the reasons for the negative findings observed in our study are not known, it could be due to difference in pathogenesis between MCI and epilepsy-specific cognitive impairment.

## 5 Limitations

Our study has limitations. The analysis is based on cross-sectional design and a small sample size of 74 PWE. Due to small sample size, we could not assess cognitive impairment as per type of seizure or epilepsy. Further, neuroimaging data was not collected in the present work which could have provided a more comprehensive understanding of cognitive impairment in PWE.

## 6 Conclusion and implications

To sum up, our study was the first to evaluate the relationship between MoCA and BDNF in adult PWE in India and Worldwide. Our data suggests that the decreased MoCA score corresponds to the reduced serum BDNF levels. MoCA total and its domains including visuospatial and executive function, naming, attention, language, abstraction, delayed recall, and orientation scores were lowered in the cognitively impaired group. This indicates that MoCA is a suitable and sensitive tool for detection of cognitive impairment in adult PWE. BDNF levels were significantly reduced in the cognitively impaired group compared to the cognitively unimpaired group. These findings point toward a possibility of BDNF as a new treatment target and clinical diagnosis for cognitive impairment in epilepsy which needs to be investigated further. Further, education was considered as a risk factor for cognitive impairment in adult PWE. Additional, longitudinal & prospective investigations are needed to validate the relationship between BDNF and MoCA score in a larger sample size of adult PWE.

## Data Availability

The original contributions presented in the study are included in the article/Supplementary material, further inquiries can be directed to the first author.

## Footnotes

^1^ https://mocacognition.com/
