# Supplementary material for: Brain-derived neurotrophic factor but not beta-secretase 1, vascular endothelial growth factor, glial fibrillary acidic protein and interleukin-1β correlate with cognitive impairment in adult persons with epilepsy: a cross-sectional single-center study from India
Source: Front Neurol. 2025 Apr 11;16:1540915. doi: 10.3389/fneur.2025.1540915 (PMC12023754; doi:10.3389/fneur.2025.1540915)
Supplement: Supplementary file 1 [file Data_Sheet_1.pdf]

Figure S1. Area under receiver operator curve (AUROC) of the total MoCA and cognitive impairment

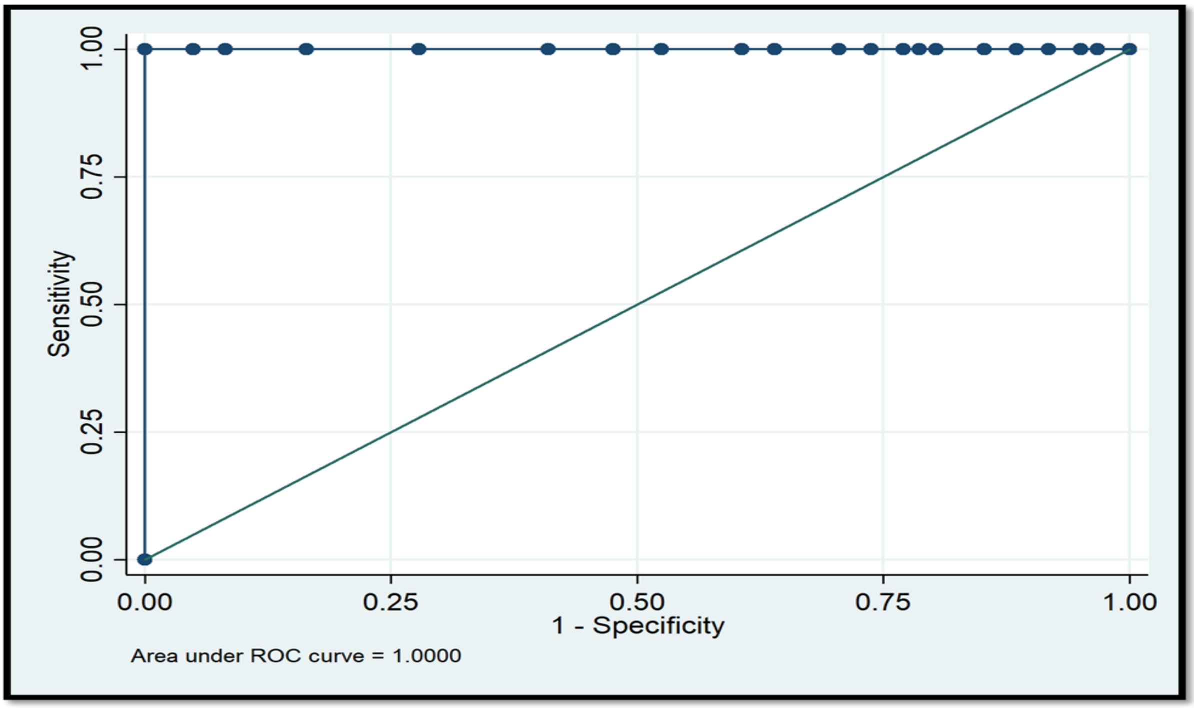

Figure S2. Area under receiver operator curve (AUROC) of BDNF and cognitive impairment

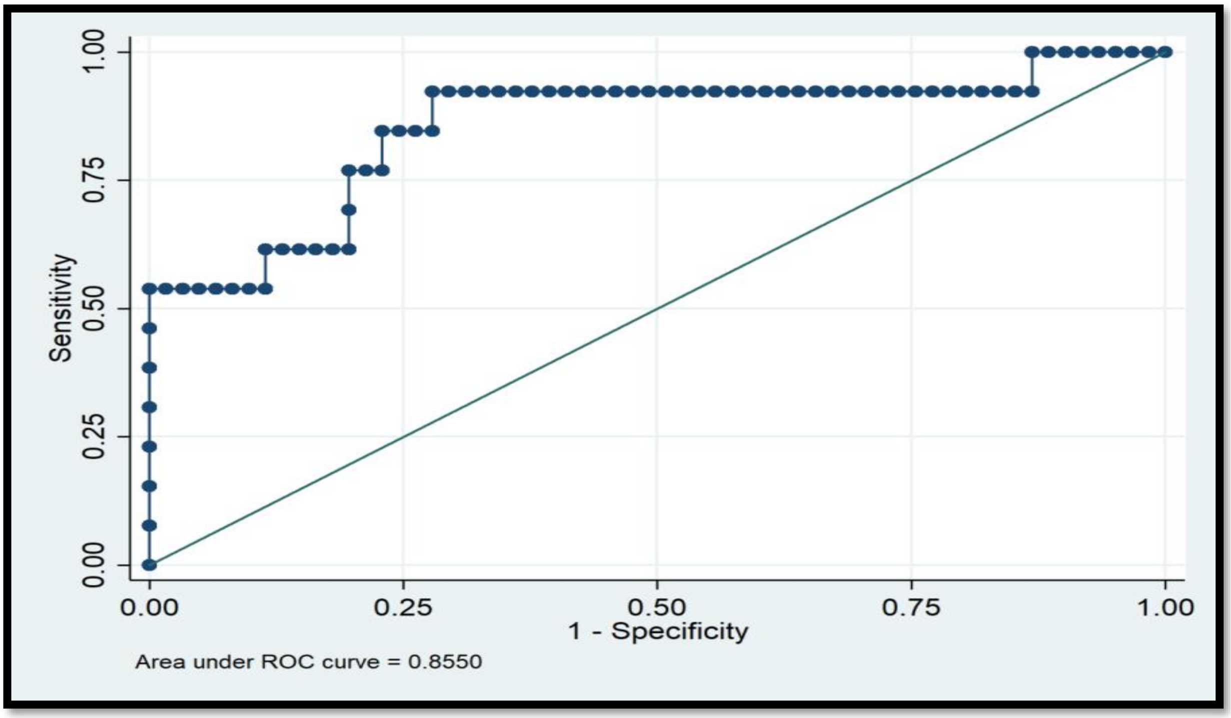

Receiver operating characteristic (ROC) curves were generated to calculate cut-off values for the various biomarkers to differentiate between cognitively impaired and unimpaired. Optimal cut-off values were measured by calculating the sensitivity and specificity of the various biomarkers at various cut-off points. Youden index was calculated to find out the associated criterion with maximum sensitivity and specificity for predicting cognition.

Figure S3. Area under receiver operator curve (AUROC) of the GFAP and cognitive impairment

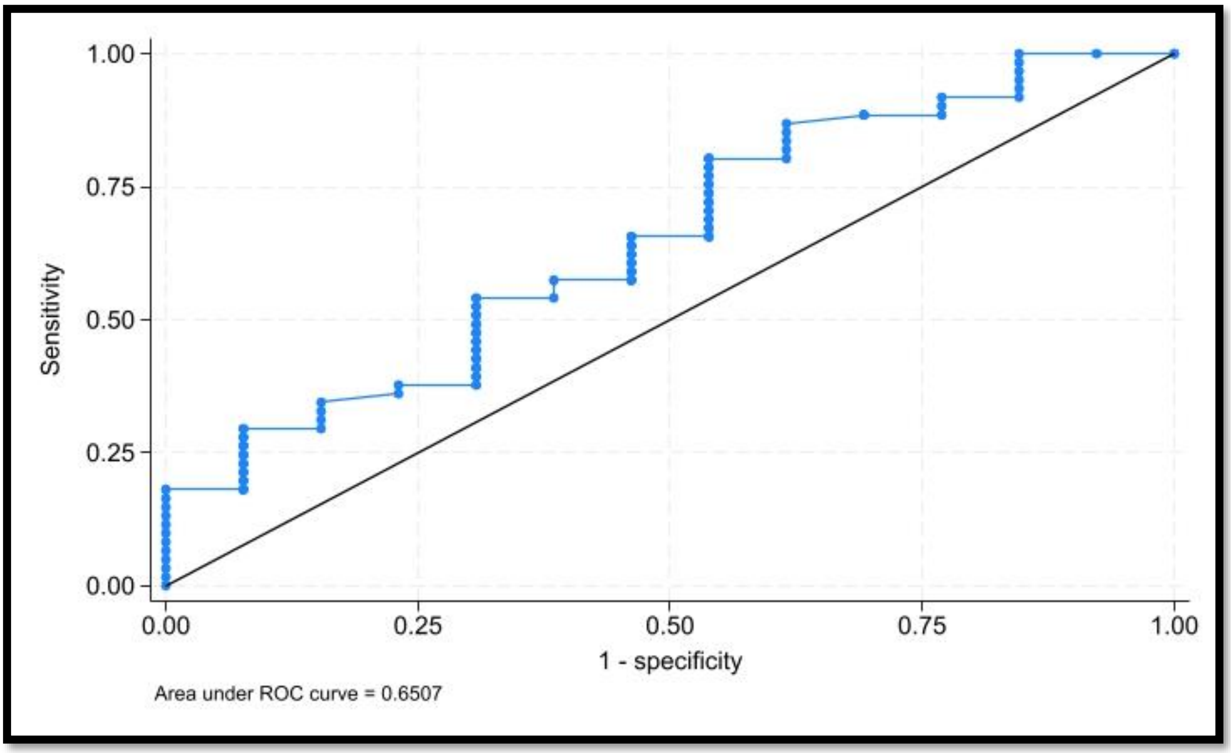

Figure S4. Area under receiver operator curve (AUROC) of the BACE-1 and cognitive impairment

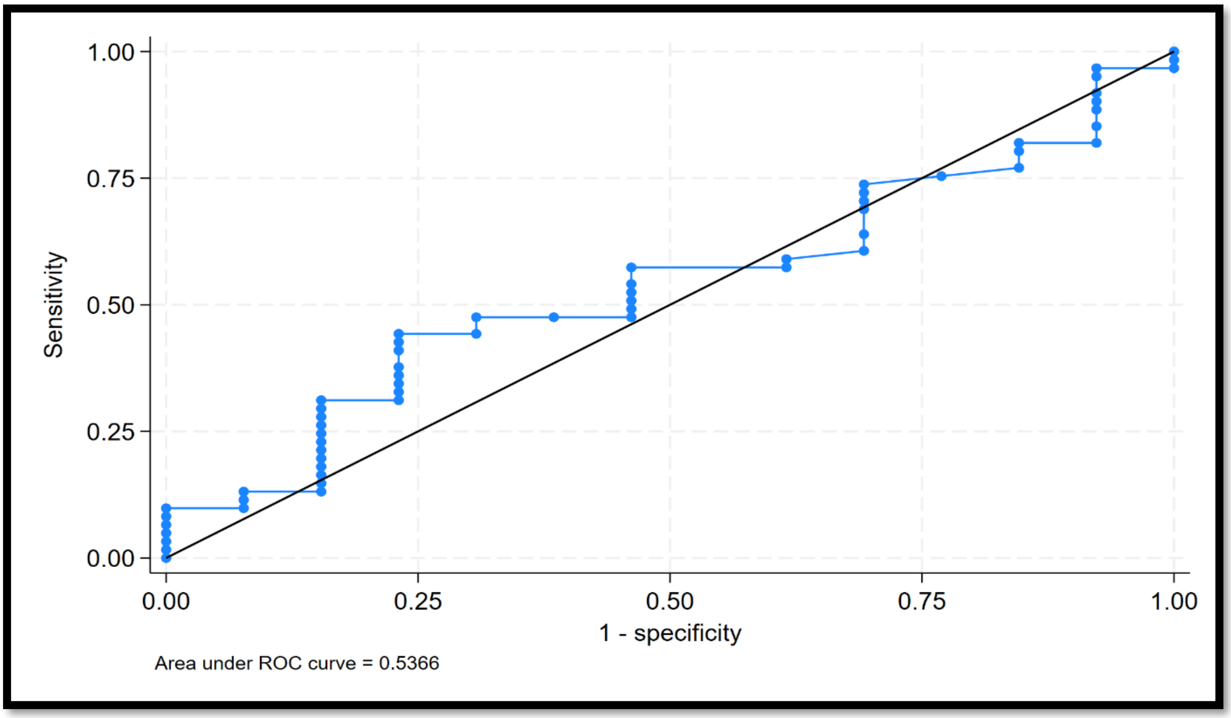

Figure S5. Area under receiver operator curve (AUROC) of VEGF and cognitive impaiment

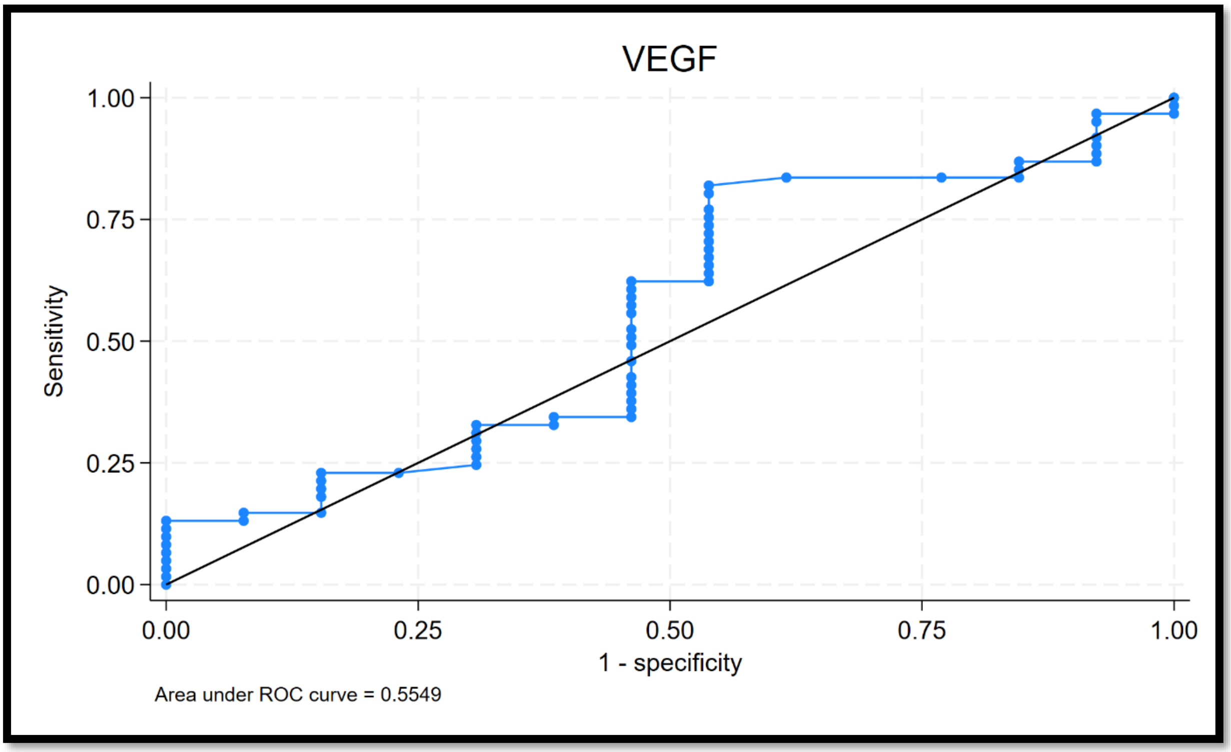

Figure S6. Area under receiver operator curve (AUROC) of IL-1β and cognitive impairment

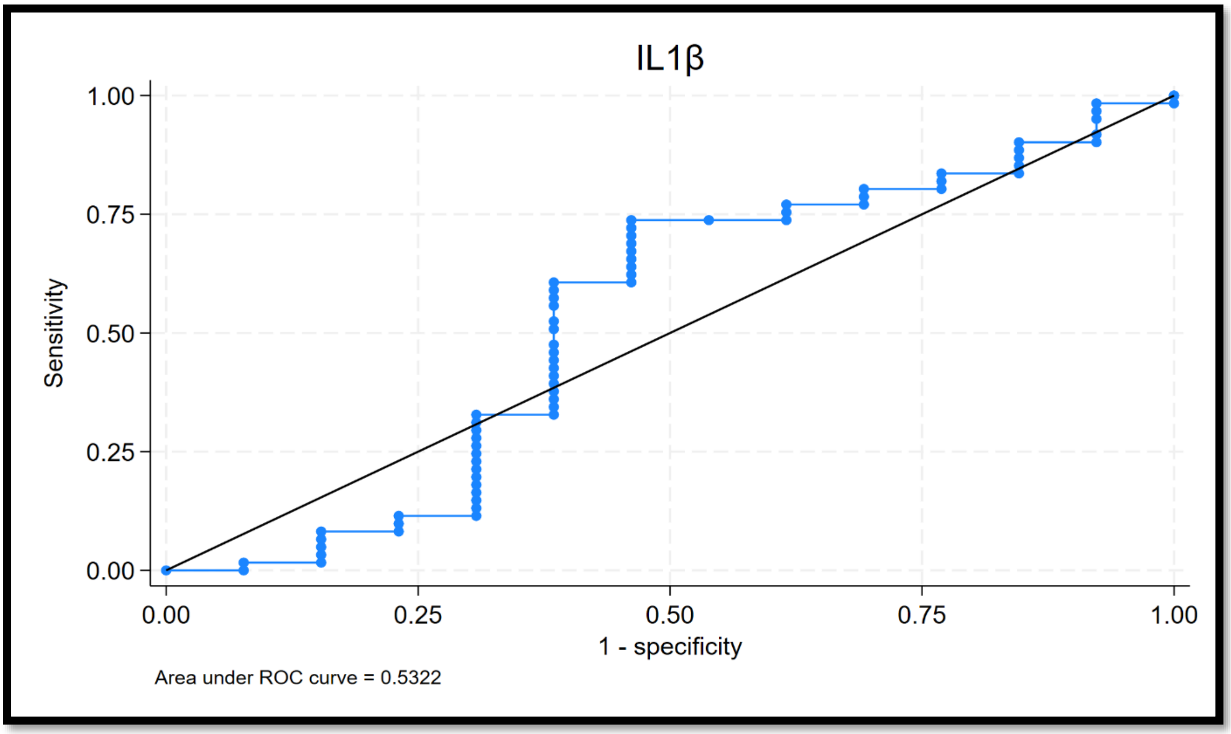

Figure S7. Montreal Cognitive Assessment Scale (MoCA) 7.1 version

MONTREAL COGNITIVE ASSESSMENT (MOCA)

Version 7.1 Original Version

NAME :
Education : Date of birth :
Sex : DATE :

			POINTS
**VISUOSPATIAL / EXECUTIVE**	Copy cube	Draw CLOCK (Ten past eleven) (3 points)	
[ ]	[ ]	[ ] Contour [ ] Numbers [ ] Hands	__/5

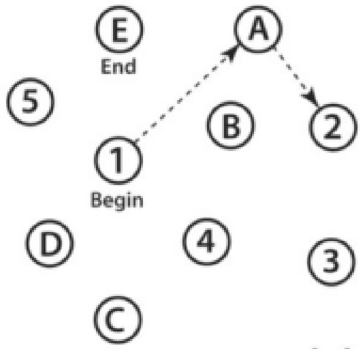

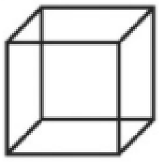

NAMING

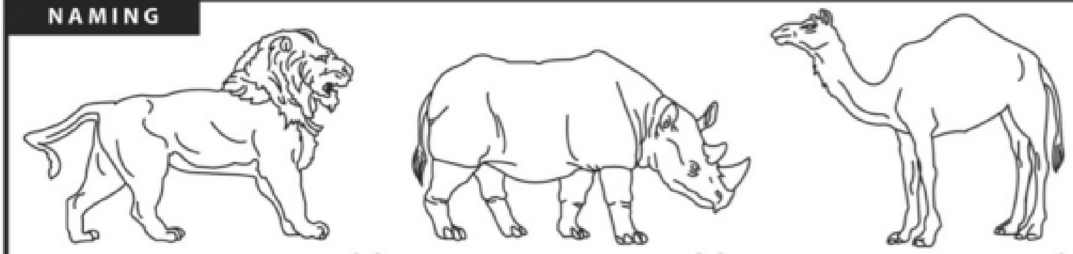

[ ] [ ] [ ] __/3

MEMORY Read list of words, subject must repeat them. Do 2 trials, even if 1st trial is successful. Do a recall after 5 minutes.

	FACE	VELVET	CHURCH	DAISY	RED
1st trial					
2nd trial					

No points

ATTENTION Read list of digits (1 digit/ sec.). Subject has to repeat them in the forward order [ ] 2 1 8 5 4
Subject has to repeat them in the backward order [ ] 7 4 2 __/2

Read list of letters. The subject must tap with his hand at each letter A. No points if ≥ 2 errors
[ ] F B A C M N A A J K L B A F A K D E A A A J A M O F A A B __/1

Serial 7 subtraction starting at 100 [ ] 93 [ ] 86 [ ] 79 [ ] 72 [ ] 65
4 or 5 correct subtractions: **3 pts**, 2 or 3 correct: **2 pts**, 1 correct: **1 pt**, 0 correct: **0 pt** __/3

LANGUAGE Repeat : I only know that John is the one to help today. [ ]
The cat always hid under the couch when dogs were in the room. [ ] __/2

Fluency / Name maximum number of words in one minute that begin with the letter F [ ] _____ (N ≥ 11 words) __/1

ABSTRACTION Similarity between e.g. banana - orange = fruit [ ] train – bicycle [ ] watch - ruler __/2

**DELAYED RECALL**	Has to recall words WITH NO CUE	FACE [ ]	VELVET [ ]	CHURCH [ ]	DAISY [ ]	RED [ ]	Points for UNCUED recall only	__/5
**Optional**	Category cue							
	Multiple choice cue							

ORIENTATION [ ] Date [ ] Month [ ] Year [ ] Day [ ] Place [ ] City __/6

© Z.Nasreddine MD www.mocatest.org Normal ≥ 26 / 30 TOTAL __/30

Administered by: ______________ Add 1 point if ≤ 12 yr edu

Table S1. Classification of therapy based on number of patients.

**Monotherapy**	**Number of patients**
Levetiracetam	1
Phenytoin	3
Carbamazepine	1
Levetiracetam	2
Clobazam	2
Valproic acid	2
Oxcarbazepine,	1
**Polytherapy**	**Number of patients**
Levetiracetam, lacosamide	1
Levetiracetam, clobazam, carbamazepine	2
Levetiracetam, clobazam, oxcarbazepine	1
Levetiracetam, oxcarbazepine	1
Levetiracetam, clobazam	2
Levetiracetam, divalproex	1
Clobazam, phenytoin	2
Phenytoin, clobazam, levetiracetam	1
Phenytoin, clobazam	1
Clobazam, levetiracetam, lacosamide, valproic acid	1
Valproic acid, lacosamide, oxcarbazepine	1
Lacosamide, clobazam,	1
Lacosamide, carbamazepine, valproic acid, sodium valproate	1
Levetiracetam, valproic acid, clobazam	1
Levetiracetam, clonazepam	1
Phenytoin, sodium valproate	1
Levetiracetam, lamotrigine, oxcarbazepine, lacosamide, sodium valproate, valproic acid, clobazam	1
Levetiracetam, lacosamide, clobazam, valproic acid, carbamazepine	1
Levetiracetam, lacosamide, clobazam, topiramate	1
Levetiracetam, clobazam, clonazepam	1
Levetiracetam, lacosamide, clobazam	1
Carbamazepine, clobazam	1
Levetiracetam, perampanel, clobazam, topiramate	1
Lamotrigine, levetiracetam, clobazam, valproic acid	1
Levetiracetam, clobazam, oxcarbazepine, lacosamide,	1
Levetiracetam, clobazam, phenytoin, divalproex sodium	1
Clobazam, lamotrigine, zonisamide, oxcarbazepine, brivaracetam	1
Levetiracetam, carbamazepine, clobazam	1
Carbamazepine, lacosamide, clonazepam	1
Lacosamide, levetiracetam, clobazam, valproic acid, perampanel	1
Valproic acid, carbamazepine	1
Levetiracetam, clobazam, carbamazepine	1
**Levetiracetam, clobazam, oxcarbazepine**	**4**
Sodium valproate, valproic acid,	1
Levetiracetam, clobazam, oxcarbazepine, topiramate	1
**Clobazam, valproic acid**	**5**
Clobazam, zonisamide, brivaracetam	1
Phenytoin, lacosamide, clobazam, levetiracetam, phenobarbitone	1
oxcarbazepine, Sodium valproate	1
Phenytoin, valproic acid, clobazam, levetiracetam, sodium valproate	1
Levetiracetam, clobazam	1
Clobazam, brivaracetam	1
Lacosamide, brivaracetam	1
Levetiracetam, clobazam, carbamazepine, perampanel	1
**Levetiracetam, clobazam**	**4**
Levetiracetam, carbamazepine	1
Lacosamide, sodium valproate	1
Valproic acid, clobazam, oxcarbazepine	1
Levetiracetam, clobazam, carbamazepine	1

Table S2. Most commonly drug prescribed in polytherapy.

Anti-seizure medications	No. of patients
Clobazam	36
Levetiracetam	33
Valproic acid	23
Lacosamide	15
Carbamazepine	12
Oxcarbazepine	9
Phenytoin	8
Brivaracetam	4
Lamotrigine	3
Perampanel	3
Clonazepam	3
Topiramate	3
Zonisamide	2
Phenobarbitone	1

Table S3. Standard calibration range, Sensitivity and Specificity of ELISA kits as per manufacturer protocol.

Human ELISA	Standard Calibration Range	Sensitivity	Specificity
BDNF pg/ml	31.25pg/ml – 2000 pg/ml	30 pg/ml	The antibodies used in the kit for capture and detection are monoclonal antibodies for human BDNF.
BACE-1 ng/ml	0.156 ng/ml – 10 ng/ml	0.061 pg/ml	The assay has high sensitivity and excellent specificity for the detection of BACE-1.
VEGF pg/ml	15.6 pg/ml – 1000 pg/ml	6.0 pg/ml	The assay has high sensitivity and excellent specificity for detection of VEGF.
IL-1β pg/ml	3.9 pg/ml – 250 pg/ml	3 pg/ml	The antibodies used in the kit for capture and detection are monoclonal antibodies for human IL-1β.
GFAP ng/ml	0.156 ng/ml – 10 ng/ml	0.056 ng/ml	The assay has high sensitivity and excellent specificity for the detection of GFAP.
